# Sputum quality and diagnostic performance of GeneXpert MTB/RIF among smear-negative adults with presumed tuberculosis in Uganda

**DOI:** 10.1371/journal.pone.0180572

**Published:** 2017-07-07

**Authors:** Amanda J. Meyer, Collins Atuheire, William Worodria, Samuel Kizito, Achilles Katamba, Ingvar Sanyu, Alfred Andama, Irene Ayakaka, Adithya Cattamanchi, Freddie Bwanga, Laurence Huang, J. Lucian Davis

**Affiliations:** 1 Department of Epidemiology of Microbial Diseases, Yale School of Public Health, New Haven, Connecticut, United States of America; 2 Department of Health Sciences & Special Education, Africa Renewal University, Kampala, Uganda; 3 Department of Medicine, Mulago Hospital, Makerere University, Kampala, Uganda; 4 Clinical Epidemiology Unit, Makerere University, Kampala, Uganda; 5 Infectious Diseases Research Collaboration, Mulago Hospital, Kampala, Uganda; 6 Division of Pulmonary and Critical Care Medicine, University of California San Francisco, San Francisco, California, United States of America; 7 Department of Microbiology, Makerere University, Kampala, Uganda; 8 Division of HIV, Infectious Diseases, and Global Medicine, University of California San Francisco, San Francisco, California, United States of America; 9 Pulmonary, Critical Care, and Sleep Medicine Section, Yale School of Medicine, New Haven, Connecticut, United States of America; Fundació Institut d’Investigació en Ciències de la Salut Germans Trias i Pujol, Universitat Autònoma de Barcelona, SPAIN

## Abstract

**Background:**

Introduction of GeneXpert MTB/RIF (Xpert) assay has constituted a major breakthrough for tuberculosis (TB) diagnostics. Several patient factors may influence diagnostic performance of Xpert including sputum quality.

**Objective:**

We carried out a prospective, observational, cross-sectional study to determine the effect of sputum quality on diagnostic performance of Xpert among presumed TB patients in Uganda.

**Methods:**

We collected clinical and demographic information and two sputum samples from participants. Staff recorded sputum quality and performed LED fluorescence microscopy and mycobacterial culture on each sample. If both smear examinations were negative, Xpert testing was performed. We calculated diagnostic yield, sensitivity, specificity, and other indicators for Xpert for each stratum of sputum quality in reference to a standard of mycobacterial culture.

**Results:**

Patients with salivary sputum showed a trend towards a substantially higher proportion of samples that were Xpert-positive (54/286, 19%, 95% CI 15–24) compared with those with all other sputum sample types (221/1496, 15%, 95% CI 13–17). Blood-stained sputum produced the lowest sensitivity (28%; 95% CI 12–49) and salivary sputum the highest (66%; 95% CI 53–77). Specificity didn’t vary meaningfully by sample types. Salivary sputum was significantly more sensitive than mucoid sputum (+13%, 95% CI +1 to +26), while blood-stained sputum was significantly less sensitive (-24%, 95% CI -42 to -5).

**Conclusions:**

Our findings demonstrate the need to exercise caution in collecting sputum for Xpert and in interpreting results because sputum quality may impact test yield and sensitivity. In particular, it may be wise to pursue additional testing should blood-stained sputum test negative while salivary sputum should be readily accepted for Xpert testing given its higher sensitivity and potentially higher yield than other sample types. These findings challenge conventional recommendations against collecting salivary sputum for TB diagnosis and could inform new standards for sputum quality.

## Introduction

Introduction of the GeneXpert MTB/RIF (Xpert) assay has constituted a major breakthrough for tuberculosis (TB) diagnostics, providing a rapid and accurate way of identifying TB patients in high TB-burden, low-income countries [1, 2]. Nevertheless, post-implementation studies have identified several challenges [3–6], emphasizing the need for deeper understanding of clinical and operational factors affecting real-world performance [7]. Previous studies have shown that pauci-bacillary forms of TB are more commonly identified in patients who are HIV-seropositive [8–10] and in those who are sputum acid-fast bacilli (AFB) smear-negative, and may reduce the overall sensitivity of Xpert in reference to the standard of mycobacterial culture [11–13]. However, because microscopy is also less sensitive in these populations, these groups are also the ones most likely to benefit from Xpert, and in whom Xpert has been especially recommended [14, 15].

A recent systematic review identified no studies describing the effect of sputum quality on Xpert performance [16, 17]. This is surprising because international guidelines have long emphasized macroscopic sputum quality as an important determinant of performance of smear microscopy and culture [16, 18]. Furthermore, previous small studies have shown salivary samples may be unsuitable for Xpert testing [19, 20]. Therefore, we sought to determine the effect of sputum quality on the diagnostic performance of Xpert in a large cohort of AFB smear-negative, presumed pulmonary TB patients in Kampala, Uganda.

## Methods

### Study population

From September 2008 through January 2016, we carried out a prospective, observational, cross-sectional study to determine the effect of sputum quality on diagnostic accuracy of Xpert. This study was carried out at Mulago National Referral Hospital, an inpatient tertiary-care facility affiliated with Makerere University in Kampala, Uganda. We enrolled consecutive adults with possible pulmonary TB into the Mulago Inpatient Non-invasive Diagnosis of Pneumonia—International HIV-associated Opportunistic Pneumonia study, as previously described [21–23]. Patients with cough ≥2 weeks but <6 months were presumed to have TB. For this sub-study, we included the subset of patients with ≥2 negative and no positive sputum AFB-smear examinations by fluorescence microscopy. We excluded participants with sputum collected via induction and those missing Xpert or culture results. Xpert results indeterminate after two tests were considered missing.

### Procedures

Following written informed consent, parent study participants provided clinical and demographic information and two expectorated sputum samples collected one hour apart. Trained research staff delivered standardized instructions on proper sputum submission [24]. Laboratory technicians graded quality of each specimen as blood-stained, mucoid, purulent, or salivary, using standardized photographs from International Union Against TB and Lung Disease guidelines [18]. Technicians examined smears via fluorescence microscopy, and if both were negative, they performed direct Xpert testing on the second sample. Staff interpreted sputum quality prior to microscopy and Xpert testing and were therefore blinded to results. Separate laboratory technicians at the Uganda National TB Reference Lab performed mycobacterial culture on Lowenstein-Jensen solid media on two sputum specimens for each patient, as previously described [21]. Finally, consenting HIV-infected smear- and Xpert-negative individuals without medical contraindication underwent diagnostic bronchoscopy with bronchoalveolar lavage, with fluid sent for concentrated AFB-smear microscopy and culture and other microbiologic assays, as previously described [25].

### Statistical analysis

We performed univariate analyses of participant characteristics, and bivariate analyses stratified by sputum quality type. We compared dichotomous variables using chi-squared tests, and continuous variables using the Wilcoxon rank-sum test. We calculated simple diagnostic yield as the proportion of each specimen type that were Xpert-positive. We also calculated sensitivities, specificities, positive and negative predictive values, and positive and negative likelihood ratios for Xpert for each stratum of sputum quality in reference to a gold standard of mycobacterial culture on two sputum samples and, if available, on bronchoalveolar lavage. We compared diagnostic yield by specimen type for our primary analysis. As a secondary analysis, we also compared the sensitivities and specificities of samples of different sputum quality types to confirm that differences in yield reflected differences in true-positive results. We selected the comparisons of diagnostic yield for the primary analysis because this metric reflects how treatment decisions are guided in routine practice. Another reason for this choice was that diagnostic sensitivity, the usual standard metric for comparisons of performance, may have limitations for the current analysis because sputum characteristics may reduce the yield of both the index test and the reference test, sputum culture, leading to uncertain effects on diagnostic accuracy. Finally, we conducted a multivariate analysis adjusting for age, gender, HIV status, CD4 count, cigarette smoking, and alcohol use, in order to assess the extent to which differences in performance reflect differences in patient characteristics versus differences in sputum characteristics. Although sample size was based on convenience, we calculated 95% confidence intervals for all study measures. We performed all analyses using STATA version 14.1 (Stata Corporation, College Station, Texas).

### Human subjects

The Makerere School of Medicine Research Ethics Committee, the Uganda National Council for Science and Technology, the Mulago Hospital Institutional Review Board, the University of California San Francisco Committee on Human Research, and the Yale Human Research Protection approved the study.

## Results

### Study population

**Fig 1:**
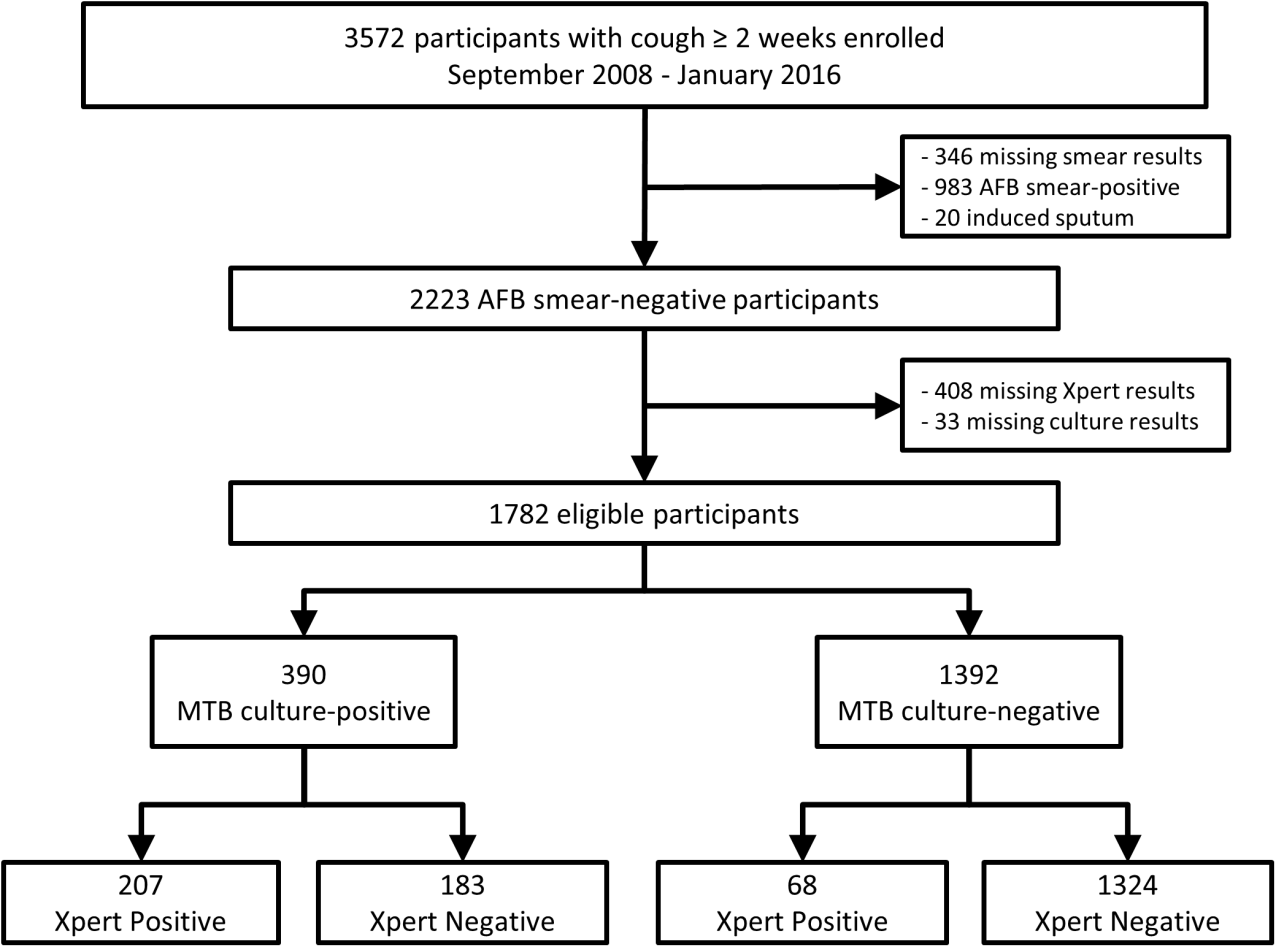
Study population. Abbreviations: AFB, acid-fast bacilli; MTB, Mycobacterium tuberculosis; Xpert, Xpert MTB/RIF assay.

Of 3572 patients enrolled in the parent study from September 2008 through January 2016, 1782 (50%) were eligible for this analysis (Fig 1). Of those ineligible, 983 were smear positive (28%), 346 were missing AFB-smear results (10%), 20 had sputum collected via induction (0.6%), 408 had missing Xpert results (11%), and 33 had missing culture results (1%). There were six time-periods when Xpert was not performed due to technical problems, accounting for most (92%) of the missing Xpert data. Eighty-nine (4%) of 2223 smear-negative patients had one indeterminate Xpert result and 14 (0.7%) remained indeterminate upon repeat. An additional four (0.2%) failed to have a second Xpert test performed after the indeterminate result, resulting in 18 (1%) with unobtainable Xpert test results.

Patients were generally young, with median age 34 years (inter-quartile range 28–44; Table 1). Most participants were men (51%). A majority were HIV-infected (66%). Of those with HIV, a majority (68%) had CD4 counts ≤200 cells/μL. Only 212 participants (12%) had a previous history of TB. While only 26% of participants had smoked more than 99 cigarettes in their lifetimes, a much higher proportion had ever drunk alcohol (65%).

**Table 1:** Clinical and demographic characteristics.

Characteristic	All Patients	Salivary	Non-Salivary	p-Value^A^
		Sputum Sample	Sputum Sample	
	(n = 1782)	(n = 286)	(n = 1496)	
**Median age** (IQR), years	34 (28–44)	34 (28–43)	34.5 (28–44)	0.78^B^
**Women (%)**	868 (49)	165 (58)	703 (47)	0.001
**Previous TB history (%)**	212 (12)	29 (10)	183 (12)	0.32
**HIV seropositive (%)**	1170 (66)	183 (64)	987 (66)	0.52
**CD4 count** ≤ 200 cells/μL^C^	796 (69)	140 (78)	656 (67)	0.005
**Tobacco smoker**^D^ (%)	467 (26)	66 (23)	401 (27)	0.19
**Previous alcohol consumption**	1158 (65)	172 (60)	986 (66)	0.061
**MTB culture positive**	390 (22)	70 (25)	320 (21)	0.25

**Abbreviations:** IQR, Inter-quartile range; TB, tuberculosis; MTB, *Mycobacterium tuberculosis*

**Legend:**

^A^ Pearson chi-squared p-value utilized unless noted

^B^ Wilcoxon rank-sum test utilized for age p-value

^C^ Only for those who are HIV-seropositive; 13 responses missing

^D^ Defined as those who have smoked more than 99 cigarettes in their lifetime

### Sputum quality

A majority of samples provided were mucoid (1296, 73%), with salivary being the next most common sample type (286, 16%). Blood-stained (119, 7%) and purulent (81, 4%) samples were less common. When comparing patients producing salivary sputum with those producing other sputum types, there were few statistically significant differences. Women, however, were significantly more likely to produce salivary sputum than men (19% vs. 13%, Risk Ratio (RR) 1.44 95% Confidence Interval (CI) 1.16–1.78, p = 0.001). While there was no significant association between sputum type and HIV status, HIV-infected patients with CD4 counts >200 cells/μL were significantly less likely to produce salivary sputum than those with CD4 counts ≤200 cells/μL (11% vs. 18%, RR 0.63 95% CI 0.45–0.88, p = 0.005).

### Diagnostic performance

Of 1782 smear-negative patients, 390 (22%) had positive *Mycobacterium tuberculosis* (MTB) culture results, while 1392 (78%) had negative MTB cultures (Fig 1). Among MTB culture-positive patients, 207 had true-positive and 183 false-negative Xpert results. Among MTB culture-negative patients, 1324 had true-negative and 68 false-positive Xpert results.

**Fig 2:**
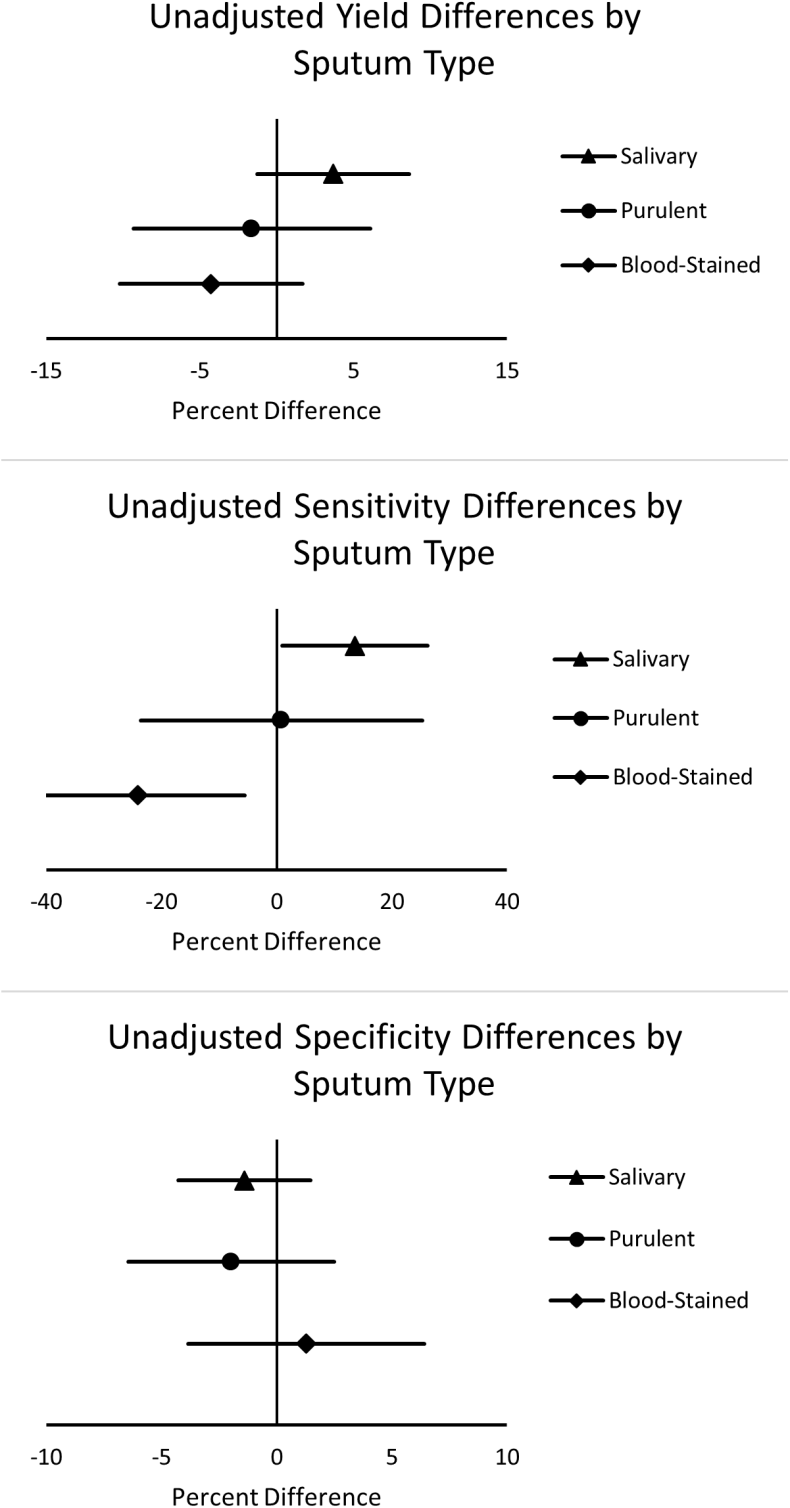
Xpert proportion positive differences and unadjusted differences in sputum Xpert sensitivity and specificity, stratified by sputum quality*. Legend: * Mucoid sputum utilized as the reference group.

Patients with salivary sputum had a substantially higher proportion of samples that were Xpert-positive (54/286, 19%, 95% Confidence Interval (CI) 15–24) compared with those with all other sputum sample types (221/1496, 15%, 95% CI 13–17), yielding 4% (95% CI -0.8 to 9, p = 0.08) more TB diagnoses. There were no significant differences between the proportions positive for each sample type when compared to mucoid sputum (Fig 2). We saw a similar proportion of MTB culture-positive results among those with salivary sputum (70/286, 25%, 95% CI 20–30) as among those with all other sample types (320/1496, 21%, 95% CI 19–24), arguing against a significant effect of specimen type on the yield of culture. Furthermore, we found that those with positive Xpert results had higher semi-quantitative results on solid culture media than those with negative results; this association did not significantly differ in comparing those with salivary specimens to those with non-salivary specimens.

The overall diagnostic sensitivity of Xpert was 53% (95% CI 48–58), and the overall, specificity was 95% (95% CI 94–96; Table 2). Specificity did not vary meaningfully among sample types, with similar results for blood-stained (94%; 95% CI 87–98), mucoid (95%; 95% CI 93–96), salivary (96%; 95% CI 93–98), and purulent samples (97%; 95% CI 89–100); none of the differences in specificity by sputum type were clinically or statistically significant (Fig 2). We also considered whether the effect of a prior diagnosis of TB on the frequency of false positive Xpert results differed by specimen type. Among those with salivary sputum, none of the eight (0%) patients with false positive results had a history of prior TB while among those with non-salivary sputum, nine of 60 (15%) did (Difference 15%, 95% CI -6.0 - + 24, p = 0.24). Stratified by sputum sample type, Xpert sensitivity varied in a manner that reproduced the differences in positive yield reported above, with blood-stained sputum identifying the lowest proportion (28%; 95% CI 12–49) and salivary sputum producing the highest proportion (66%; 95% CI 53–77) of TB patients. Xpert sensitivity was similar among purulent and mucoid specimens, with a difference of +1% (95% CI -23 to +25, p = 0.95; Fig 2). However, salivary sputum was 13% (95% CI +1 to +26, p = 0.04) more sensitive than mucoid sputum, and blood-stained sputum was 24% (95% CI -42 to -5, p = 0.02) less sensitive than mucoid sputum. Positive likelihood ratios (LR+) varied by sputum type, with blood-stained sputum providing the weakest LR+ (4.4; 95% CI 1.6–12) and salivary the strongest LR+ (17.7; 95% CI 8.8–36). Negative likelihood ratios were similar for salivary (0.36; 95% CI 0.26–0.49), mucoid (0.50; 95% CI 0.45–0.57), and purulent (0.49; 95% CI 0.29–0.81) sputum types, while blood-stained sputum had the weakest negative likelihood ratio (0.77; 95% CI 0.60–0.99).

**Table 2:** Diagnostic performance of GeneXpert among 1782 study participants, stratified by sputum quality characteristics.

	All	Blood-stained	Mucoid	Purulent	Salivary
	Samples	Sputum	Sputum	Sputum	Sputum
				
	n = 1782	n = 119	n = 1296	n = 81	n = 286
	(95% CI)	(95% CI)	(95% CI)	(95% CI)	(95% CI)
Sensitivity, %	53 (48–58)	28 (12–49)	52 (46–58)	53 (28–77)	66 (53–77)
Specificity, %	95 (94–96)	94 (87–98)	95 (93–96)	97 (89–100)	96 (93–98)
PPV, %	75 (70–80)	54 (25–81)	74 (67–80)	82 (48–98)	85 (73–93)
NPV, %	88 (86–90)	83 (75–90)	88 (86–90)	89 (79–95)	89 (85–93)
LR+	10.9 (8.5–13.9)	4.4 (1.6–11.9)	10.2 (7.7–13.6)	16.9 (4.0–71.2)	17.7 (8.8–35.8)
LR-	0.49 (0.44–0.55)	0.77 (0.60–0.99)	0.50 (0.45–0.57)	0.49 (0.29–0.81)	0.36 (0.26–0.49)

**Abbreviations:** CI, Confidence Interval; PPV, Positive Predictive Value; NPV, Negative Predictive Value; LR+, Positive Likelihood Ratio; LR-, Negative Likelihood Ratio.

**Legend:** Below are the counts in each diagnostic accuracy category, with a single sputum Xpert as the index test, and mycobacterial culture on at least two sputum samples and bronchoalveolar lavage (if available) as the reference standard test

*All samples*: 207 true positives, 183 false negatives, 68 false positives, 1324 true negatives.

*Blood-stained*: 7 true positives, 18 false negatives, 6 false positives, 88 true negatives.

*Mucoid*: 145 true positives, 133 false negatives, 52 false positives, 966 true negatives.

*Purulent*: 9 true positives, 8 false negatives, 2 false positives, 62 true negatives.

*Salivary*: 46 true positives, 24 false negatives, 8 false positives, 208 true negatives.

After adjusting for age, HIV status, CD4 count, gender, smoking, and alcohol use, the overall effect of sputum quality on Xpert sensitivity remained significant (p = 0.006), without meaningful changes in the above-reported sensitivity differences. Adjusted sensitivity of salivary sputum remained significantly different from mucoid samples (p = 0.02), as did adjusted sensitivity of blood-stained sputum (p = 0.01).

## Discussion

Specimen quality has long been assumed to be as an important predictor of the performance characteristics of microbiologic tests, particularly those used to diagnose lower respiratory-tract infections. Unfortunately, the amount and quality of evidence about how sputum quality affects the performance of TB diagnostic tests is limited. In this prospective cross-sectional study, we found no significant difference in diagnostic yield of Xpert testing between salivary and non-salivary specimens among adults with negative sputum AFB-smear examinations in a low-income country with high burdens of TB and HIV. In fact, we identified a strong trend towards a higher diagnostic yield in salivary than in non-salivary specimens. These differences were confirmed by a secondary comparison of diagnostic accuracy in reference to mycobacterial culture. This analysis showed significantly higher diagnostic sensitivity of Xpert on salivary samples as compared with the referent category, mucoid sputum samples, while blood-stained sputum was associated with significantly lower sensitivity.

Macroscopic quality has long been emphasized in guidelines on the use of smear microscopy in TB evaluation. Despite this emphasis, there is only one published study of sputum quality and smear microscopy, which demonstrated substantially higher sensitivity with purulent or bloody sputum as compared with mucoid or salivary sputum among 170 TB patients [20]. However, 40% had culture-negative TB, and it is unclear if these associations apply equally to patients with microbiologically confirmed TB, or be relevant to less pauci-bacillary populations. While a recent systematic review found no studies on the influence of sputum quality on the performance of Xpert [16], we identified two subsequently published studies addressing this question. One compared diagnostic sensitivity and specificity by sputum type among 136 culture-confirmed TB patients and 703 culture-negative non-TB patients in Kenya, but there were only a few modest differences by sample type and none of these reached statistical significance [19]. The second study enrolled over 21,000 household contacts in Vietnam but almost all samples collected were mucoid, which prevented meaningful comparisons with other sample types [17].

Thus, our finding that salivary sputum does not have lower but perhaps higher diagnostic yield when testing for TB with Xpert may have great clinical importance. Salivary sputum has been considered unsuitable for examination by smear microscopy, and therefore laboratory staff have historically been trained to discourage patients from producing and submitting salivary sputum samples in preference for other sample types. Our results suggest that the conventional assumptions that salivary sputum is of lower quality and bloody sputum of higher quality for smear microscopy do not apply to samples tested with Xpert. Given the observational study design, we were not able to explore reasons why salivary sputum may provide greater sensitivity than other samples. Potential reasons could include: a greater bacillary load of MTB DNA in saliva than in other sample types; a greater recovery of MTB DNA from salivary sputum than from more viscous sample types; or more efficient amplification of MTB DNA from saliva than from samples such as sputum that have a more complicated specimen matrix that could include inhibitors of amplification. The last explanation is unlikely because the Xpert assay includes a positive control to detect inhibitors in all samples.

Our findings about the enhanced yield of salivary sputum may be of additional importance in high HIV-burden areas because of the inverse association we identified between CD4 count and the likelihood of producing salivary sputum. Because those with lower CD4 counts are also more likely to develop TB and to be smear-negative, it is crucial that they be tested with a diagnostic tool that has high sensitivity and high likelihood of obtaining a true positive result [15, 26]. Since salivary sputum had the highest diagnostic sensitivity of any specimen type tested, salivary samples should not be rejected for Xpert testing. Additional studies might explore whether saliva, particularly when obtained after coughing and prior to eating or oral care, can provide comparable diagnostic sensitivity to that of other sputum types [27].

In contrast, blood-stained sputum appears less desirable for Xpert testing in smear-negative populations. Xpert testing of blood-stained sputum missed twice as many TB cases as it diagnosed. A potential explanation for lower sensitivity could be that blood is a known inhibitor of DNA amplification, although this is less likely because by design Xpert inhibition should be detected by failed amplification of the internal positive control and reported as “Invalid”[28]. Nevertheless, our results do raise concern about the suitability of bloody sputum for molecular testing, and merit further investigation to identify the mechanisms underlying the low sensitivity. Until then, practitioners may consider attempting to obtain a non-bloody sample if a bloody specimen tests Xpert-negative.

Our study had some limitations. First, a majority of samples were mucoid (73%), resulting in small sample sizes for other sputum types and relatively large confidence intervals for all study measures. In particular, our primary analysis comparing diagnostic yield by specimen type is underpowered, because the 95% confidence intervals for yield differences include clinically important effects. Nevertheless, our sub-analyses were sufficiently powered to detect meaningful differences in sensitivity among three of four sputum types. Second, our sub-analyses could have been biased if the yield of sputum culture is also influenced by specimen quality. However, direct comparisons showed no difference in culture yield, and even if underpowered, the similarities in effect size and direction of our yield and accuracy analyses make this unlikely. Furthermore, we may have misclassified some culture-negative TB patients as not having TB, since we utilized solid rather than liquid culture media [29]. However, since our gold standard was rigorously determined utilizing multiple mycobacterial culture samples, we believe that this misclassification is minor and would not substantially bias our estimates. We may also have occasionally misclassified sputum quality. However, we utilized well-trained laboratory technologists to assess macroscopic sputum quality and provided them with visual aids to promote accurate readings. Therefore, any misclassification would likely be non-differential and unlikely to impact our results. Finally, our study focused on sputum smear-negative patients, but Xpert is now recommended as the first-line test for TB regardless of smear-status [15]. However, in many settings, Xpert testing continues to be limited to smear-negative patients due to resource constraints.

Our study also had many strengths. First, we carried out our study in a relevant population, possible TB patients in a low-income country with a high TB burden. This helps make our results generalizable to many other populations being tested with Xpert in high-burden, resource-limited settings. Second, our study is the first of sufficient size and power to provide meaningful comparisons of the effects of sputum quality on both Xpert diagnostic accuracy and Xpert diagnostic yield. We therefore believe our study fills a crucial gap in understanding Xpert testing.

In conclusion, for patients who are smear-negative, utilizing Xpert may provide a rapid diagnosis that might have otherwise been missed. As it replaces smear microscopy in an increasing number of high-burden countries, it has the potential to reduce the time and number of visits needed to obtain a diagnosis. Our findings, however, demonstrate the need to exercise caution in collecting sputum for Xpert and in interpreting results because sputum quality may impact test yield and sensitivity differently from what has been traditionally taught for smear microscopy. In particular, it may be wise to pursue additional testing should a blood-stained sputum test negative, especially in high TB-burden communities. In addition, laboratory staff should not reject salivary sputum for Xpert testing but accept it readily given its higher sensitivity and potentially higher yield than other sample types. Future studies attempting to replicate these findings and examining additional factors that may impact Xpert diagnostic performance are warranted to help enhance the yield and sensitivity of Xpert testing.

## Supporting information

S1 File Xpert sputum quality dataset. Complete dataset utilized for data analysis.
(CSV)
